# MRIES: A Matlab Toolbox for Mapping the Responses to Intracranial Electrical Stimulation

**DOI:** 10.3389/fnins.2021.652841

**Published:** 2021-06-14

**Authors:** Kaijia Sun, Haixiang Wang, Yunxian Bai, Wenjing Zhou, Liang Wang

**Affiliations:** ^1^School of Systems Science, Beijing Normal University, Beijing, China; ^2^CAS Key Laboratory of Mental Health, Institute of Psychology, Beijing, China; ^3^Epilepsy Center, Tsinghua University Yuquan Hospital, Beijing, China; ^4^Department of Psychology, University of Chinese Academy of Sciences, Beijing, China

**Keywords:** electrical stimulation, cortico-cortical evoked potential, Epilepsy, SEEG, functional connectivity

## Abstract

**Propose:**

Directed cortical responses to intracranial electrical stimulation are a good standard for mapping inter-regional direct connectivity. Cortico-cortical evoked potential (CCEP), elicited by single pulse electrical stimulation (SPES), has been widely used to map the normal and abnormal brain effective network. However, automated processing of CCEP datasets and visualization of connectivity results remain challenging for researchers and clinicians. In this study, we develop a Matlab toolbox named MRIES (Mapping the Responses to Intracranial Electrical Stimulation) to automatically process CCEP data and visualize the connectivity results.

**Method:**

The MRIES integrates the processing pipeline of the CCEP datasets and various methods for connectivity calculation based on low- and high-frequency signals with stimulation artifacts removed. The connectivity matrices are saved in different folders for visualization. Different visualization patterns (connectivity matrix, circle map, surface map, and volume map) are also integrated to the graphical user interface (GUI), which makes it easy to intuitively display and compare different connectivity measurements. Furthermore, one sample CCEP data set collected from eight epilepsy patients is used to validate the MRIES toolbox.

**Result:**

We show the GUI and visualization functions of MRIES using one example CCEP data that has been described in a complete tutorial. We applied this toolbox to the sample CCEP data set to investigate the direct connectivity between the medial temporal lobe and the insular cortex. We find bidirectional connectivity between MTL and insular that are consistent with the findings of previous studies.

**Conclusion:**

MRIES has a friendly GUI and integrates the full processing pipeline of CCEP data and various visualization methods. The MRIES toolbox, tutorial, and example data can be freely downloaded. As an open-source package, MRIES is expected to improve the reproducibility of CCEP findings and facilitate clinical translation.

## Introduction

Mapping human brain connectivity quantitatively on a large scale has attracted increasing attention in recent years. Understanding brain connectivity enables us to explore the normal functions and dysfunctions of the brain. Numerous studies have revealed important physiological (Watrous et al., 2013; Solomon et al., 2017, 2019) and psychological evidence (van Diessen et al., 2013; Bartolomei et al., 2017; Lagarde et al., 2018) from the perspective of brain connectivity. There are many methods of probing brain connectivity, such as diffusion tensor imaging (DTI), which is used to measure anatomical connectivity, and functional magnetic resonance imaging (fMRI), a way of measuring functional connectivity. However, such neuroimaging techniques cannot quantitatively measure the information that is transferred directly between the areas. In comparison with statistical-based approaches (e.g., Granger causality) for measuring direct connectivity from the EEG or fMRI signals (Reid et al., 2019; Jafarian et al., 2020), detecting cortical responses to invasive electrical stimulation is a good standard for mapping inter-regional direct connectivity, and has been widely used in physiological (Enatsu et al., 2015, 2016; Keller et al., 2018; Usami et al., 2018; Dionisio et al., 2019) and pathological studies (David et al., 2011; van ’t Klooster et al., 2011, 2017; Boido et al., 2014; Bartolomei et al., 2017; Tousseyn et al., 2017; Zhao et al., 2019).

Single pulse electrical stimulation (SPES) has been used to elicit cortical responses since 1990 (Wilson et al., 1990, 1991). The evoked potentials recorded in other cortices are termed cortico-cortical evoked potential (CCEP). CCEP provides a way to explore functional networks in the living human brain, including language system (Matsumoto et al., 2004; Keller et al., 2011; Koubeissi et al., 2012; Saito et al., 2014; Tamura et al., 2016), motor system (Matsumoto et al., 2006; Swann et al., 2012; Terada et al., 2012; Enatsu et al., 2013), and the limbic network (Almashaikhi et al., 2014; Enatsu et al., 2015; Jiménez-Jiménez et al., 2015). With regard to epilepsy, SPES has been used for two major purposes, one to detect the epileptogenic zone, and the other to probe the epileptic network during seizures (Matsumoto et al., 2017).

There are many different methods to characterize the properties of CCEP responses. For low-frequency (LF) responses, the typically evoked waves (e.g., N1/P1) are commonly used as an indicator. The amplitude and latency of the peak are considered as the direct response strength and latency (Conner et al., 2011; Keller et al., 2011; Parker et al., 2018). The root mean square (RMS) calculated from the signals after electrical stimulation is also widely used to represent the response strength (Dionisio et al., 2019; Prime et al., 2020). Recent studies have revealed high-frequency (HF) responses to electrical stimulation in remotely recorded areas. Given that the broadband gamma activity is usually proposed to reflect local neural population activity (Crone et al., 2001; Voytek et al., 2009; Wang et al., 2010), broadband gamma activity in the recorded electrodes can be used to represent the response strength evoked by electrical stimulation (van ’t Klooster et al., 2011; Crowther et al., 2019).

Currently, the steps of CCEP data processing are cumbersome and time-consuming for many researchers and clinicians without programming experience. Few packages have been developing to process CCEP data and visualize the results. For example, the toolbox called FAST (Taylor et al., 2020) is mainly developed for visualizing the low-frequency responses at the channel level. However, this toolbox did not provide an integrated function including various calculation methods that were important for comparing different indicators in the same dataset. Furthermore, in clinical applications and basic research related to CCEP intuitively presenting the connectivity results in different ways is essential but unfortunately is lacking in the FAST toolbox. The traditionally and widely used toolboxes, such as EEGLAB (Delorme and Makeig, 2004) and Fieldtrip (Oostenveld et al., 2010), were developed for processing electrophysiological data and cannot be directly used for CCEP data.

In this study, we develop a Matlab toolbox named MRIES (Mapping the Responses to Intracranial Electrical Stimulation) to integrate the full pipeline of CCEP data processing and connectivity visualization methods. The users can easily complete the entire data processing in the GUI. Common visualization methods are also integrated in the GUI, which makes it easy to intuitively display and compare different indicators and visualization methods. To assess the validity of the toolbox, we applied it and explored direct connectivity between the medial temporal lobe and insular in eight epilepsy patients.

## Materials and Methods

### Subjects and Data Acquisition

The subjects in this study were eight patients with refractory focal epilepsy who underwent SEEG electrode implantation at the hospital. They were all implanted with SEEG electrodes to further delineate the epileptogenic zone. The clinical characteristics of eight patients are summarized in Table 1. Their age ranged from 21 to 38 years old (27.50 ± 6.12). These patients have medial temporal lobe and insular contacts: five of them have electrodes in the left hemisphere; one of them has electrodes in the right hemisphere; two of them have electrodes in both hemispheres. For analysis, we only choose the hemisphere in which there are medial temporal lobe (MTL) and insular contacts. This sample CCEP data set is used to validate the MRIES toolbox.

**TABLE 1:** Demographic information of patients for MTL-Insular connectivity analysis.

**Patient ID**	**1**	**2**	**3**	**4**	**5**	**6**	**7**	**8**
Age (year)	28	24	24	33	31	38	21	21
Gender	M	M	F	M	M	F	M	F
Implanted hemisphere	R	B	L	B	L	L	L	L
Analysis hemisphere	R	R	L	L	L	L	L	L
Number of MTL contacts	11	10	15	5	5	8	8	9
Number of Insular contacts	8	3	4	14	4	10	3	9
Number of connectivity between MTL and Insular	88	30	60	70	20	80	24	81
Number of significant connectivity MTI- > Insular	20	22	21	16	9	21	6	51
Number of significant connectivity Insular- > MTL	19	2	15	43	0	28	10	12

*M, male; F, female; R, right; L, left; B, bilateral; MTL, medial temporal lobe.*

The data was acquired using a Neurofax EEG-1200 system at a sampling rate of 2,000 Hz. The electrical stimulus consisted of a constant current square wave pulse of 0.3 ms duration, the current intensity of 5 mA, and the pulse frequency of 1 Hz with alternative polarity. About fifty biphasic pulses of electrical stimulation were delivered to two neighboring contacts in each session, while the EEG signals of all electrode contacts were recorded.

### Electrode Reconstruction and Selection

Post-implantation CT images were co-registered onto pre-implantation MR images using Freesurfer (v6.0.0,^1^). The registration was visually verified and manually adjusted if necessary. Intracranial electrodes were identified using clustering-based segmentation and classified according to anatomical landmarks in the native space (Qin et al., 2017). For visualization of all subjects’ electrodes, each subject’s MRI was co-registered to the MNI space. All electrodes were then superimposed onto the standard brain. The location of electrode contacts was ascertained by visual inspection by the neurologists (HXW and WJZ).

Cortical and volumetric parcellations were performed using the Desikan-Killiany atlas in Freesurfer. The insular contacts were selected based on their location within the insular itself, and the medial temporal contacts were selected based on their location in the hippocampus, amygdala, entorhinal, and parahippocampal gyrus. The numbers of ROI contacts from all patients are listed in Table 1.

### MRIES Processing Procedures

MRIES is a GUI-based Matlab toolbox for Windows, Mac, and Linux operation systems and can be used without Matlab programming experience. MRIES can calculate and map the responses to either a single pulse or repeated multiple electrical stimulations, including data preprocessing, response detection, connectivity matrix construction, and various visualization methods. The required input data are: (1) the recorded data of CCEP; (2) the information file, including subject ID and electrode labels that can be manually edited in a text format; (3) pre-operative T1 and post-operative CT images; (4) brain pial and parcellation files output from FreeSurfer; (5) contact coordinates file that can be manually generated. The set of the tools are easily available by Matlab-based GUI and the open-source package is suitable for customized functions and modules. It is worth noting that all parameters mentioned below can be set flexibly in a customized way. Here, we describe the detailed procedures.

**FIGURE 1:**
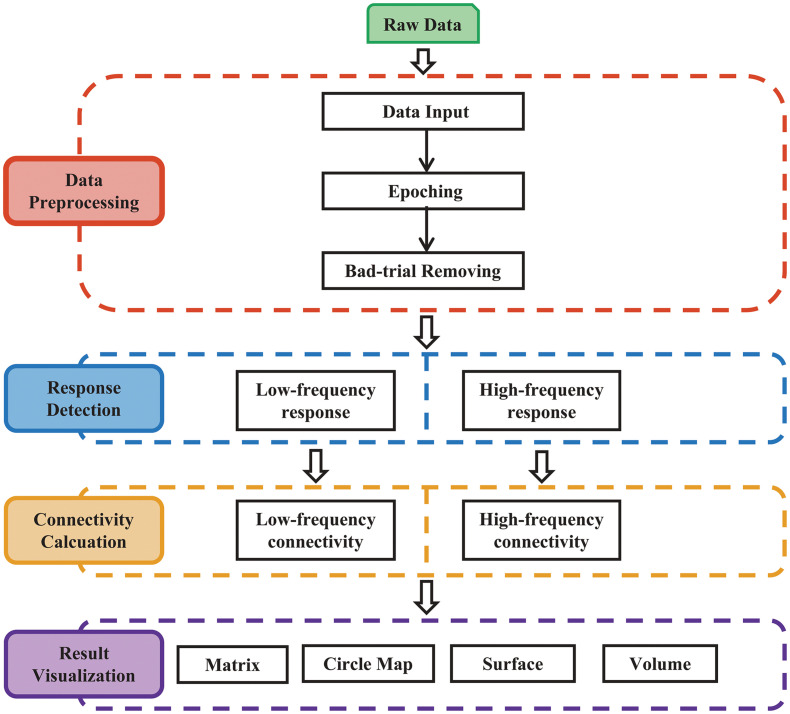
Main procedure for pipeline processing and visualization of responses to electrical stimulation. The procedure includes four parts: (1) data preprocessing; (2) response detection of electrical stimulation; (3) cortico-cortical connectivity calculation; and (4) result visualization.

The procedures of MRIES include four steps: (1) data preprocessing; (2) response detection; (3) connectivity matrix construction; and (4) connectivity visualization (Figure 1).

#### Data Preprocessing

##### Data input

The EEG files should be in European data format (edf) so that they can be converted *via* the clinical EEG recording system. The file can be sorted as the rank of the electrodes, and the invalid and unavailable channels should be removed in advance. Because electrophysiological data are always recorded with high time resolution and simultaneously recorded with multiple channels, the whole CCEP data of one subject is too big to be loaded into the memory of the computer. To solve this problem, the toolbox prefers to deal with the segmented files, which consist of the partial data by grouping some contacts (e.g., dozens of stimulation contact pairs) in succession or even one single contact pair. After this step, the edf files can be separated into the different stimulated contact pairs, renamed in the order of electrodes, and transformed to mat format for the next step.

##### Epoching

Before this step, we used a high-pass filter (>0.1 Hz) to remove linear drift. Then, the epoching step aimed to extract and align repeated trials from the completely recorded data corresponding to the same stimulated contacts (mat file generated in the “Data input” step).

The epoching processing involved two steps, detecting stimulation onset and aligning epochs. To detect stimulation onset, in some cases the stimulation events were recorded in an independent marker channel simultaneously. At the stimulation beginning, a pulse signal will be marked in that marker channel. Thus, the peaks in the marker channel are automatically detected as stimulations and the start of peaks are detected as the stimulation onset. In other cases, in which there is no specific marker channel, one needs to detect the stimulation onset *via* the artifacts in the recorded contacts induced by the electrical stimulation. In this case, the user can enter the channel number to detect the stimulation and stimulation onset.

If the number of stimulations was detected correctly, the processing then went to the aligning step. Then the specific time window before and after stimulation onset was extracted as an epoch and all epochs were aligned at stimulation onset for significant response detection.

##### Bad-epoch removal

Bad-epoch removal was based on the variation of the different epochs. The abnormal signal (e.g., epileptic discharge) causes an obvious large voltage amplitude compared with the normal signal. We used the 3 standard deviations above the mean as a threshold to remove the epochs with abnormal signals.

#### Response Detection

##### Low-frequency response

**FIGURE 2:**
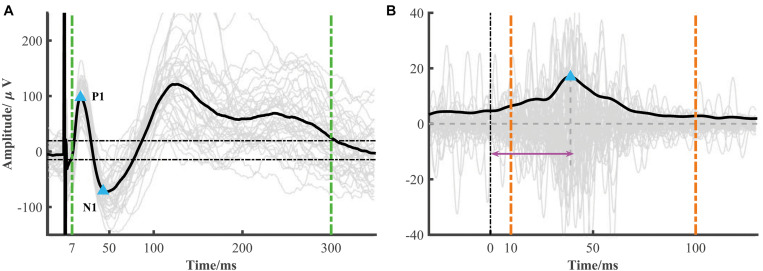
Low- and high-frequency responses extracted from a single session. **(A)** A representative curve of low-frequency response (black line) averaged from each epoch response (gray lines). The blue triangles represent significant N1 and P1 peak response which is determined by 6 standard deviations of baseline (the two horizontal dotted lines) and in the time range from 7 to 50 ms. The RMS is calculated from 7 to 300 ms after stimulation (two green vertical dotted lines). **(B)** A representative curve of high-frequency response (black line), i.e., the averaged broadband gamma envelop signal over different epochs (gray lines). The variance is calculated from the averaged gamma envelop from 10 to 100 ms after electrical stimulation (two orange vertical dotted lines). The blue triangle represents high-frequency peak response. The peak latency is marked by the pink arrow.

The typical low-frequency response consisted of an early negative or positive wave (N1/P1) and a late slow wave (N2/P2). It has been proposed that the early (or late) wave reflected a direct (or indirect) connection between the recorded and simulated location. The toolbox focused on the early response (i.e., N1 or P1). The N1 denoted the first negative wave, while the P1 was the first positive wave, and the time range of N1/P1 peak onset was limited within 7–50 ms after electrical stimulation onset to avoid the stimulation artifact. The significant peak amplitude and peak latency were used to construct the connectivity matrix (Figure 2A). The root mean square (RMS) was also widely used as an indicator of the response. We calculated the RMS of the electrode *j* based on the averaged epoch signal *x* within the time window [*t*_1_*t*_2_] after stimulation onset as follows (Figure 2A).

$$R\,M\,S_{j}=\sqrt{\frac{\sum_{t=t_{1}}^{t_{2}}x_{t}^{2}}{\left|t_{2}-t_{1}\right|}}$$

##### High-frequency response

The electrical stimulation artifacts can make a large and false activity around the stimulation onset. However, directly removing the artifacts led to discontinuous signals which introduced new artifacts. Based on the method proposed by a recent study (Crowther et al., 2019), we replaced the artifacts with the linear merged signals before and after the stimulation, extracted the broadband gamma signal (e.g., 70–170 Hz), and then computed the absolute value of the Hilbert transform of these band-pass filtered results. In our datasets, we found that the SNR (signal-to-noise) used in Crowther’s study was too strict in some response detection. Thus, instead of SNR, we used the variance as an indicator of strength. Similar to low-frequency response detection, the significant high-frequency activity and peak latency were also calculated (Figure 2B).

#### Connectivity Matrix Construction

##### Low-frequency connectivity

The low-frequency connectivity was mainly focused on response strength and latency. To detect the direct response, we chose the earlier response between N1 and P1 to perform the statistical test and extract the peak amplitude and latency as a statistical indicator of one stimulation-response pair. Given that different areas have different baseline activity, we also measured the normalized amplitude by calculating the z-score of peak amplitude by the distribution of the baseline amplitude. Thus, for one subject we can obtain the LF-amp (low-frequency amplitude), LF-lat (low-frequency latency), and LF-Zamp (low-frequency z-score amplitude) connectivity matrix. For the RMS measurement, we can obtain the LF-RMS (low-frequency RMS) connectivity matrix. We also calculated the LF-sRMS (low-frequency significant RMS) matrix by multiplying LF-RMS and binarized LF-amp.

##### High-frequency connectivity

We calculated the high-frequency connectivity by the p-value of the permutation test and also obtained the peak of the averaged high-gamma envelop signal. We used Bonferroni correction on the resultant p-values for multiple comparisons. For visualization, we calculated the negative log of the corrected p-values for the HF-str (high-frequency strength) connectivity matrix and the peak latency for the HF-lat (high-frequency latency) connectivity matrix. We also calculated the HF-sign (high frequency significant) connectivity matrix by multiplying HF-str and binarized HF-lat matrix.

#### Connectivity Visualization

##### Matrix visualization

Heat map visualization is a basic method of visualization. We use a two-dimensional heat map to represent the connectivity strength (e.g., RMS) evoked by stimulating two paired contacts.

##### Circle map visualization

We use a circle map to visualize the connectivity matrix. An anatomical atlas is required to display region information for each contact, such as the Desikan-Killiany atlas. Every node of the circle map represents one contact and the edge linking nodes represents significant connectivity and direction. The circle map offers a clear way to display the whole connectivity pattern.

##### Volume visualization

The implanted electrodes only sample part of the brain. To display the responses at a large scale, we made a linear interpolation of response around the electrode location to estimate the activity in the surrounding unsampled areas using the method proposed in a previous study (David et al., 2011). The MRIES toolbox can present the whole-brain interpolated responses in the individual brain.

##### Surface visualization

For directly displaying the connectivity and contact location in the brain surface, it is possible to show the connectivity matrix in a three-dimensional brain surface with contact locations. This method requires the brain pial and parcellation files output from FreeSurfer and contact coordination file. This visualization can intuitively display the responses in a different region.

### Statistical Analysis

#### Statistical test in response detection

Low-frequency response is detected at each time point based on the averaged signal over different epochs. The signal after the stimulation is tested at each time point. The signals after the stimulation beyond 6 standard deviations of the mean obtained from the baseline (200 ms before stimulation onset) were detected as significant time points. The significance duration was restricted, continuously lasting for more than 5 ms.

Using the method proposed by a recent study (Crowther et al., 2019), we calculated the variance of the averaged broadband gamma signal at each location between 10 and 100 ms post-stimulation. To test whether the corresponding variance value was significant, we applied a randomization test in which we repeated the variance calculation 10,000 times after reversing the broadband signals in each trial and applying a circular shift at a randomly selected interval. This produced a distribution of variance values that represented no significant modulation of broadband gamma activity in the post-stimulus period. We determined the statistical significance of post-stimulus broadband gamma changes at each electrode by applying the observed variance value to a normal cumulative distribution function calculated from the permutation distribution of log-normalized variance values for each channel. Finally, we measured the magnitude of broadband gamma modulation by calculating the negative log of the Bonferroni-corrected p-values. To establish the peak latency of the broadband gamma response, we modeled average broad-band gamma amplitudes in the 200 ms prior to a stimulus with a normal distribution and then determined the peak time after the stimulus at which the average amplitude exceeded the voltage corresponding to α = 0.001 and was the maximum within 10–100 ms.

#### Statistical analysis in the toolbox validation

We focus on direct connectivity between the medial temporal lobe and insular to validate the CCEP results output from the toolbox. The Binomial test was employed to determine the significance of inter-regional connectivity for each patient. Specifically, within the total connectivity (i.e., all pairs of stimulating and responding sites between two regions) we counted how many pairs are significant in the low-frequency connectivity detected by MRIES. Then using the significance level for each pair (i.e., the 6 standard deviations), we obtained the p value in the Binomial test. Finally, we reported the percentage of patients with significant connectivity between regions.

## Results

### The MRIES Toolbox

**FIGURE 3:**
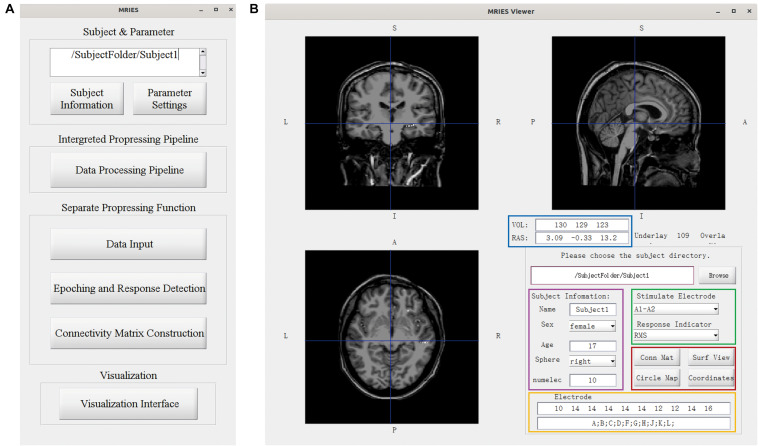
Main GUI of the toolbox. **(A)** Data processing GUI, including the processing pipeline, separate processing step, and visualization. **(B)** Visualization GUI. The individual brain is shown in different views. The lower right part shows the information and controllers highlighted in the color-coded box. The volume and voxel coordinates are illustrated in the blue box. The subject information is illustrated in the pink box. The electrode information is illustrated in the yellow box. The specific stimulated electrode pair and indicator illustrated in the green box can be chosen and then the responses to this electrode pair are shown in the volume space. Other visualization GUIs and 3D-electrode locations are shown in the red box.

The MRIES toolbox mainly included two parts, the data processing GUI (Figure 3A) and visualization GUI (Figure 3B). Before the data processing, the user needs to place the data and information files in specific folders. Then, they should press the “Subject Information” and “Parameter Setting” buttons in the data processing GUI (Figure 3A) and enter the subject, electrode, and parameter information in the newly opened window (Supplementary Figures 1, 2).

**FIGURE 4:**
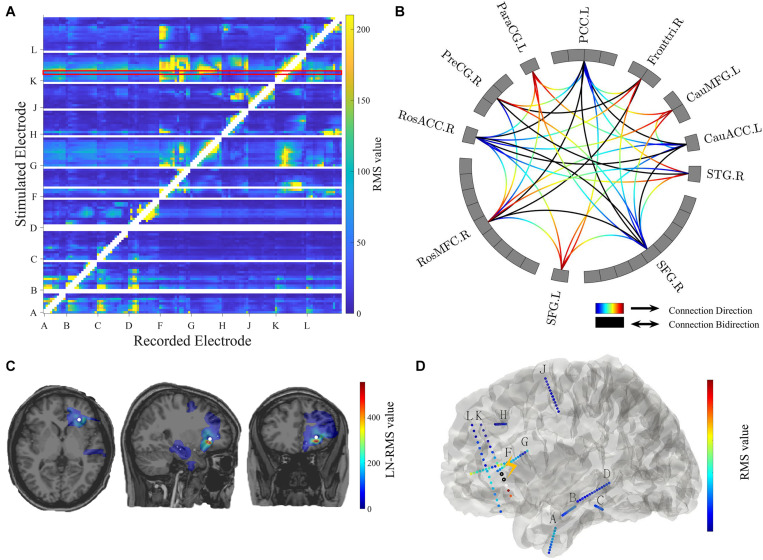
Connectivity visualization. **(A)** Connectivity matrix. The stimulated electrodes are shown in the y-axis and the recorded electrodes are shown in the x-axis. The color-coded numbers represent RMS values here, which depends on the selection of response indicators in the main GUI toolbox. The unstimulated pairs and recorded electrodes with a short distance (radius < 5 mm) to the stimulation pair are illustrated in white. The exampled stimulation result marked in the red box is shown in panels **(C,D)**. **(B)** Circle map visualization. The number of rectangular boxes in each compartment represents the number of contacts in each region labeled aside (L = left, R = right, RosACC = rostral anterior cingulate cortex, RosMFG = rostral middle frontal gyrus, SFG = superior frontal gyrus, STG = superior temporal cortex, CauACC = cauda lanterior cingulate cortex, CauMFG = caudal middle frontal gyrus, Fronttri = parstriangularis of the inferior cortex, PCC = posterior cingulate cortex, ParaCG = paracentral gyrus, PreCG = precentral gyrus). The curves represent significant connectivities. The gradient color curves represent directional connectivity (from blue to red) and the black color curves represent bi-directional connectivity. **(C)** Brain response mapping is shown in individual volume space in a format of linear interpolation surrounding the implanted electrodes. The white dot indicates stimulation position. **(D)** Surface view. All contacts and electrode labels are shown in the 3D surface view and the color represents the RMS value. The black circle dots represent stimulation contact-pairs.

In the data processing GUI, the toolbox can easily run the processing pipeline as described above to get a different connectivity matrix by pressing the button “Data Processing Pipeline.” Alternatively, the data processing GUI can also be used separately for specific processing steps. Pressing the button “Data input” can complete the step “*Data Input.”* Pressing the button “Epoching and Response Detection” can complete the steps “*Epoching*,” “*Bad-epoch removal*,” and “*Response detection.*” Pressing the button “Connectivity matrix construction” can complete the step “*Connectivity Matrix Construction.*” In both completely separated situations, the data will be processed automatically and the process progress will show in command after pressing this button. After the data processing, the user can press the “Visualization Interface” button to open the visualization GUI (Figure 3B). Different visualization methods are integrated into the Visualization GUI (Figure 4). In these GUIs, we can also add some controllers to display patient information and change the connectivity matrix and stimulation contacts (for volume and surface view GUI). The detailed processing steps and GUIs can be seen in the tutorial.

#### Matrix GUI

The controller “Conn Mat” in visualization GUI (Figure 3B) can open the matrix GUI. There are two parts in this GUI: the matrix visualization and control area. The matrix visualization is the heat map of the connectivity matrix (Figure 4A). Color represents the value of the connectivity matrix, and the abscissa and ordinate represent stimulation and response contacts. The control area includes the sliders, which are used to ignore the closer response contact and lower response value, and the display boxes to display the stimulation contact, response contact, and response value.

#### Circle Map GUI

The circle map GUI shows the circle map of the connectivity matrix described above. As shown in Figure 4B, every rectangular box in the circle represents a contact, and the region label is shown outside the circle. The curve represents significant connectivity. The gradient color curves represent direct connection and the black color curves represent the bi-direction connection.

#### Volume GUI

The volume GUI, integrated into the main visualization GUI (Figure 3B), includes two parts, the displaying area, and the control area. The displaying area can display the individual brain structure. After choosing specific stimulation contact and connectivity matrix, the volume visualization also can be displayed in the brain (Figure 4C). The control area can input the patient folder, choose the specific stimulation contact-pair and connectivity matrix, and open the other visualization GUI. These controllers can update the whole visualization.

#### Surface GUI

The surface GUI also includes the visualization area and control area. All contacts and electrode labels are shown in the surface view and the color represents the response value. The black circle dots represent stimulation contact-pairs (Figure 4D). In the control area, there are buttons that rotate the visual angle of the surface brain. The information of the stimulation contact is also displayed if contact information is input in the MRIES.

### Data Organization

Each patient should have a unique folder, and the input and output files are under this folder and organized as below.

#### Input Data

The input data consists of five parts. (1) The recorded data of CCEP converted to edf format and placed in the *edf* folder, and the relevant information about the edf file is entered into the file *edf_match.txt*. (2) Patient information: subject and electrode information are entered into the file *subjectinfo.txt*. (3) Reconstruction results: one can copy Freesurfer reconstruction result to the subject folder. (4) Pre-operative T1 and post-operative CT images: the two images should be co-registrated first and placed in the *brain3D* folder. (5) Contact coordinate file: the coordinates of contacts are saved into the file *autocoordinate.mat* and placed in the *brain3D* folder. Detailed input data organization can be seen in the tutorial.

#### Output Result

For each subject, the MRIES toolbox automatically generates some file folders to store the result. Specifically, the *subj_elec_info.mat* saves the patient and electrode information. The *data* folder includes the mat file of every contact pair converted from edf files, and each line of one file in *data* means the repeat trials recorded in the channel. Every file in the *stimulationdata* folder includes all response indicators from repeat trails of the same stimulation contact-pairs. The *Matrix* folder includes all the connectivity matrix of all kinds of indicators, which are used in the visualization GUIs. For example, “*conn_matrix_LF_lat.mat*” means the low-frequency latency connectivity. The detailed organization of outputs can be seen in the tutorial.

### Toolbox Validation Using MTL and Insular Connectivity

**FIGURE 5:**
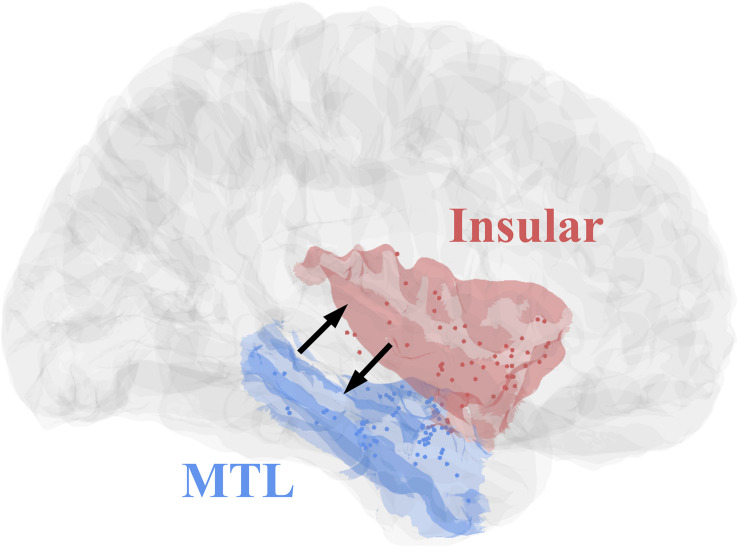
Contact distributions and direct connectivity between MTL and insular, revealed by CCEP. The contacts’ distributions of patients (Table 1) in MNI152 standard space (Insular, red; MTL, blue). The two black arrows indicate significant bidirectional connectivity between the insular and medial temporal lobe based on the result of this study.

All eight patients had significant connectivity from MTL to insular (all *p* values < 0.001, Binomial-test, Table 1). Seven of the eight patients had significant connectivity from insular to MTL (all *p* values < 0.001 except for patient 5, Binomial-test, Table 1). This bidirectional connectivity (Figure 5) between MTL and insular is consistent with the findings reported in previous studies (Enatsu et al., 2015; Dionisio et al., 2019).

## Discussion

The toolbox MRIES integrates the processing pipeline and visualization of cortical responses to electrical stimulation. Many measurements (e.g., low-frequency response and peak latency, RMS, high-frequency response, and peak latency) are calculated which makes it possible to compare the different results at the same time. Various visualization methods are also integrated into this GUI, which makes it intuitive when displaying a connectivity matrix constructed by different measurements.

The main advantage of MRIES is easy and convenient to use, allowing one to choose the full processing pipeline once the required data and information files are provided correctly. The remaining procedures can be completed automatically. If only part steps of the pipeline are needed, one can choose specific steps and obtain intermediate documents. The visualization controllers are also integrated into GUI and it is easy to further expand them.

Mapping cortical responses to electrical stimulation in the human brain is of great significance for clinical and scientific purposes. The electrical stimulation technique has been widely used for studying normal brain connectivity (Enatsu et al., 2016; Dionisio et al., 2019) and the influence of brain diseases on functional brain network (Kobayashi et al., 2017; Tousseyn et al., 2017; Davis et al., 2018). The existing toolboxes are not suitable for processing CCEP data and displaying the connectivity results. For example, although EEGLAB (Delorme and Makeig, 2004) and FieldTrip (Oostenveld et al., 2010) offer some functions for scalp EEG signal processing, they are not applied to CCEP data. In contrast, the FAST graph (Taylor et al., 2020) presents a new framework for calculating low-frequency responses to electrical stimulation and displays the results at the contact level. However, it remains difficult to carry out the calculations of various indicators and intuitive visualization of inter-regional connectivity. The MRIES toolbox was developed to address these issues and can be directly used for clinical doctors to probe the epileptic network during a seizure. The connectivity results calculated by MRIES can be easily pooled together over the subjects from multiple epilepsy centers, furthering understanding of functional brain organization.

In the current study, we also performed the validation of the MRIES toolbox based on the connectivity between the medial temporal lobe and insular. This connectivity has been widely studied in different ways, such as DTI (Ghaziri et al., 2018; Nomi et al., 2018), fMRI (Ambrosi et al., 2017), and meta-analytic (Cauda et al., 2012). Previous studies also used CCEP data to detect the connectivity between the limbic system and insular and found significant bidirectional connectivity between the two regions (Enatsu et al., 2015; Dionisio et al., 2019). We measured the CCEP connectivity between the two regions in eight epilepsy patients by MRIES and also found significant bidirectional connectivity. These consistent results provide the reliability of the MRIES.

There are several limitations to the toolbox. Currently, it only supports the European data format for raw CCEP data. Other data formats, such as Brain Imaging Data Structure (BIDS), will be integrated in the future. Some data processing steps are still time-consuming because of the limited speed of Matlab scripts. The parallel computing and C-language modules will be integrated into the MRIES toolbox to improve computational efficiency in the future.

In summary, the MRIES toolbox can auto-process the cortical responses to electrical stimulation, and include different visualization methods. The functions developed in the MRIES toolbox have been integrated into the GUI, which is convenient for researchers and clinicians without programming experience. The MRIES toolbox, tutorial, and one example data can be freely downloaded at GitHub^2^ and FigShare^3^. As an open-source package, MRIES is expected to improve the reproducibility of CCEP findings and facilitate clinical translation.

## Data Availability Statement

The original contributions presented in the study are included in the article/Supplementary Material, further inquiries can be directed to the corresponding author/s: LW, lwang@psych.ac.cn.

## Ethics Statement

The studies involving human participants were reviewed and approved by Institutional Review Board of the Institute of Psychology, Chinese Academy of Sciences. The patients/participants provided their written informed consent to participate in this study. Written informed consent was obtained from the individual(s) for the publication of any potentially identifiable images or data included in this article.

## Author Contributions

KS and LW designed the software. KS, YB, and LW developed the software. KS, HW, and LW wrote the manuscript. HW and WZ provided the experimental data. All authors contributed to the article and approved the submitted version.

## Conflict of Interest

The authors declare that the research was conducted in the absence of any commercial or financial relationships that could be construed as a potential conflict of interest.

## Footnotes

1 https://surfer.nmr.mgh.harvard.edu/

2 https://github.com/SunKaijia0065/MRIES

3 https://figshare.com/articles/dataset/MRIES_s_sample_data/14376110
