# Supplementary figures and images for: MRIES: A Matlab Toolbox for Mapping the Responses to Intracranial Electrical Stimulation
Source: Front Neurosci. 2021 Jun 14;15:652841. doi: 10.3389/fnins.2021.652841 (PMC8236813; doi:10.3389/fnins.2021.652841)

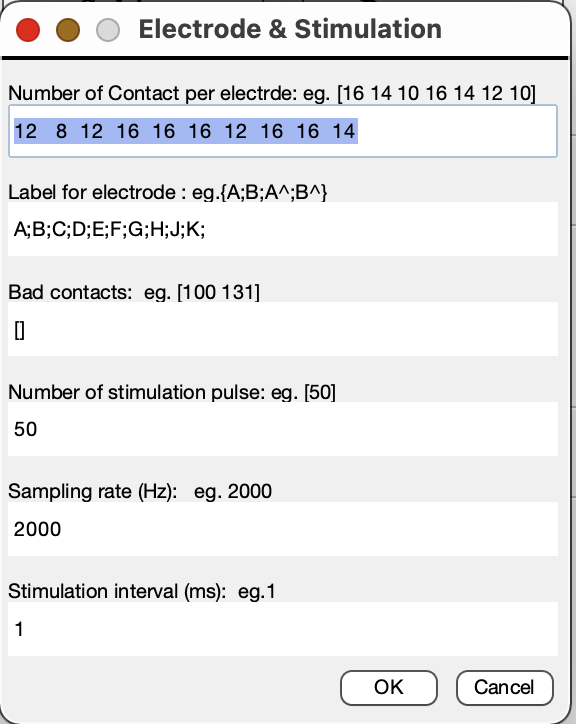

Supplement: Supplementary file 1 [file Image_1.PNG]

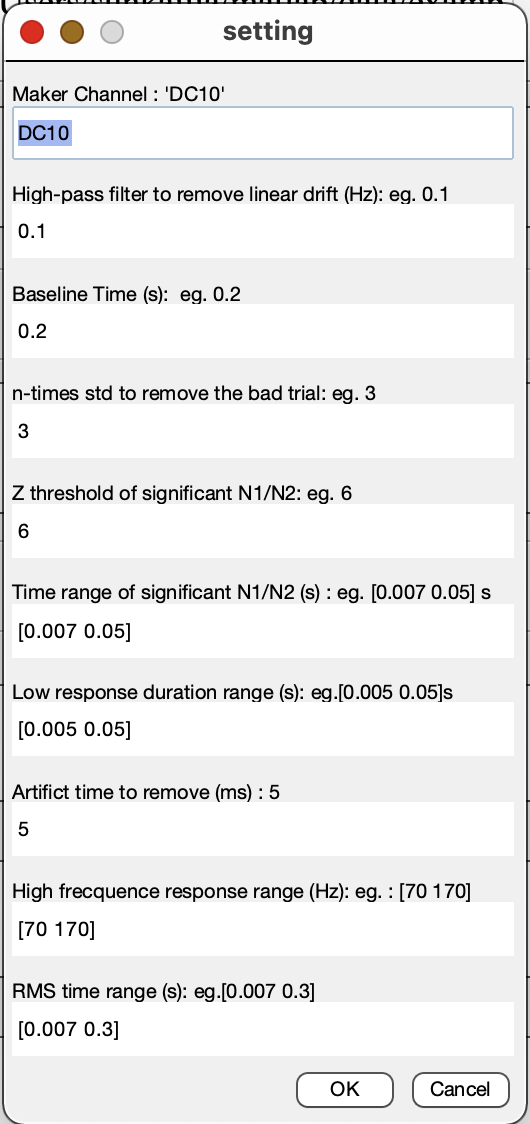

Supplement: Supplementary file 2 [file Image_2.PNG]
